# Invalid Claims About the Validity of Implicit Association Tests by Prisoners of the Implicit Social-Cognition Paradigm

**DOI:** 10.1177/1745691621991860

**Published:** 2021-03-12

**Authors:** Ulrich Schimmack

**Affiliations:** Department of Psychology, University of Toronto Mississauga

**Keywords:** implicit attitudes, Implicit Association Test, validity, prejudice, suicide, mental health

## Abstract

In a prior publication, I used structural equation modeling of multimethod data to examine the construct validity of Implicit Association Tests. The results showed no evidence that IATs measure implicit constructs (e.g., implicit self-esteem, implicit racial bias). This critique of IATs elicited several responses by implicit social-cognition researchers, who tried to defend the validity and usefulness of IATs. I carefully examine these arguments and show that they lack validity. IAT proponents consistently ignore or misrepresent facts that challenge the validity of IATs as measures of individual differences in implicit cognitions. One response suggests that IATs can be useful even if they merely measure the same constructs as self-report measures, but I find no support for the claim that IATs have practically significant incremental predictive validity. In conclusions, IATs are widely used without psychometric evidence of construct or predictive validity.

Greenwald and colleagues (1998) introduced Implicit Association Tests (IATs) as a new method to measure individual differences in implicit cognitions. Twenty years later, IATs are widely used for this purpose, but their construct validity has not been established. Even its creator is no longer sure what IATs measure. Whereas Banaji and Greenwald (2013) confidently described IATs as “a method that gives the clearest window now available into a region of the mind that is inaccessible to question-asking methods” (p. xiii), they now claim that IATs merely measure “the strengths of associations among concepts” (Cvencek et al., 2020, p. 187). This is akin to saying that an old-fashioned thermometer measures the expansion of mercury: It is true, but it has little to do with thermometers’ purpose of measuring temperature.

Fortunately, we do not need Greenwald or Banaji to define the constructs that IATs are supposed to measure. Twenty years of research with IATs makes it clear what researchers believe they are measuring with IATs. A self-esteem IAT is supposed to measure implicit self-esteem (Greenwald & Farnham, 2000). A race IAT is supposed to measure implicit prejudice (Cunningham et al., 2001), and a suicide IAT is supposed to measure implicit suicidal tendencies that can predict suicidal behaviors above and beyond self-reports (Kurdi et al., 2021). The empirical question is whether IATs are any good at measuring these constructs. I concluded that most IATs are poor measures of their intended constructs (Schimmack, 2021). This conclusion elicited one implicit and two explicit responses.

## Implicit Response

The implicit response is to simply ignore criticism and to make invalid claims about the construct validity of IATs (Greenwald & Lai, 2020). For example, a 2020 article coauthored by Nosek, Greenwald, and Banaji (among others) claimed that “available evidence for validity of IAT measures of self-esteem is limited (Bosson et al., 2000; Greenwald & Farnham, 2000), with some of the strongest evidence coming from empirical tests of the balance-congruity principle” (Cvencek et al., 2020, p. 191). This statement is as valid as Donald Trump’s claim that an honest count of votes would make him the winner of the 2020 election. Over the past 2 decades, several articles have concluded that self-esteem IATs lack validity (Buhrmester et al., 2011; Falk et al., 2015; Walker & Schimmack, 2008). It is unscientific to omit these references from a literature review.

The balance-congruity principle is also not a strong test of the claim that the self-esteem IAT is a valid measure of individual differences in implicit self-esteem. In contrast, the lack of convergent validity with informant ratings and even other implicit measures of self-esteem provides strong evidence that self-esteem IATs are invalid (Bosson et al., 2000; Falk et al., 2015).

Finally, supporting evidence is surprisingly weak. For example, Greenwald and Farnham’s (2000) highly cited article tested predictive validity of the self-esteem IAT with responses to experimentally manipulated successes and failures (*n* = 94). They did not even report statistical results. Instead, they suggested that even nonsignificant results should be counted as evidence for the validity of the self-esteem IAT:

Although *p* values for these two effects straddled the *p* = .05 level that is often treated as a boundary between noteworthy and ignorable results, any inclination to dismiss these findings should be tempered by noting that these two effects agreed with prediction in both direction and shape. (Greenwald & Farnham, 2000, p. 1032)

Twenty years later, this finding has not been replicated, and psychologists have learned to distrust *p* values that are marginally significant (Benjamin et al., 2018; Schimmack, 2012, 2020). In conclusion, conflict of interest and motivated biases undermine the objectivity of Greenwald and colleagues in evaluations of IATs’ validity.

## Explicit Response 1

Vianello and Bar-Anan (2021) criticized my structural equation models of their data. They also presented a new model that appeared to show incremental predictive validity for implicit racial bias and implicit political orientation. I thought it would be possible to resolve some of the disagreement in a direct and open communication with the authors because the disagreement is about modeling of the same data. I was surprised when the authors declined this offer, given that Bar-Anan coauthored an article that praised the virtues of open scientific communication (Nosek & Bar-Anan, 2012). Readers therefore have to reconcile conflicting viewpoints for themselves. To ensure full transparency, I published syntax, outputs, and a detailed discussion of the different modeling assumptions on OSF at https://osf.io/wsqfb/.

In brief, a comparison of the models shows that mine is more parsimonious and has better fit than their model. Because the model is more parsimonious, better fit cannot be attributed to overfitting of the data. Rather, the model is more consistent with the actual data, which in most sciences is considered a good reason to favor a model. Vianello and Bar-Anan’s model also produced unexplained, surprising results. For example, the race IAT has only a weak positive loading on the IAT method factor, and the political-orientation IAT even has a moderate negative loading. It is not clear how a method can have negative loadings on a method factor, and Vianello and Bar-Anan provided no explanation for this surprising finding.

The two models also produce different results regarding incremental predictive validity (Table 1). My model shows no incremental predictive validity for implicit factors. It is also surprising that Vianello and Bar-Anan found incremental predictive validity for voting behaviors, because the explicit and implicit factors correlated (*r*) at .9. This high correlation leaves little room for variance in implicit political orientation that is distinct from political orientation measured with self-ratings. In conclusion, Vianello and Bar-Anan failed to challenge my conclusion that implicit and explicit measures measure mostly the same constructs and that low correlations between explicit and implicit measures reflect measurement error rather than some hidden implicit processes.

**Table 1.** Predictive Validity Estimates of Race and Political Evaluations

Criterion and predictor	Vianello & Bar-Anan^a^			Schimmack		
	β	*SE*	*p*	β	*SE*	*p*
Contact						
Explicit race	−0.24	0.05	< .001	−0.49	0.16	.002
Implicit race	−0.12	0.06	.019	0.09	0.18	.588
Voting intentions						
Explicit politics	0.56	0.13	< .001	0.88	0.09	< .001
Implicit politics	0.16	0.14	.248	−0.17	0.10	.080
Past voting						
Explicit politics	0.10	0.23	.651	0.71	0.10	< .001
Implicit politics	0.66	0.23	.005	0.11	0.11	.319

a Parameters are from my reproduced Mplus model that provides standard errors for standardized coefficients.

## Explicit Response 2

The second response (Kurdi et al., 2021) is a confusing 7,000-word article that is short of facts, filled with false claims, and requires more fact-checking than a Trump interview.

### False fact 1

The authors begin with the surprising statement that my findings are “not at all incompatible with the way that many social cognition researchers have thought about the construct of (implicit) evaluation” (p. 423). This statement is misleading. For 3 decades, social-cognition researchers have pursued the idea that many social-cognitive processes that guide behavior occur outside of awareness. For example, Nosek et al. (2011) claim “most human cognition occurs outside conscious awareness or conscious control” (p. 152) and go on to claim that IATs “measure something different from self-report” (p. 153). And just last year, Greenwald and Lai (2020) claimed that “in the last 20 years, research on implicit social cognition has established that social judgments and behavior are guided by attitudes and stereotypes of which the actor may lack awareness” (p. 419).

Social psychologists have also been successful in making the term implicit bias a common term in public discussions of social behavior. The second author, Kathy Ratliff, is director of Project Implicit, which “has a mission to develop and deliver methods for investigating and applying phenomena of implicit social cognition, including especially phenomena of implicit bias based on age, race, gender or other factors” (Kurdi et al., 2021, p. 431). It is not clear what this statement means if we do not make a distinction between traditional research on prejudice with self-report measures and the agenda of Project Implicit to study implicit biases with IATs.

In addition, all three authors have published recent articles that allude to IATs as measures of implicit cognitions. In a highly cited *American Psychologist* article, Kurdi and coauthors (2019) claim “in addition to dozens of studies that have established construct validity . . . investigators have asked to what extent, and under what conditions, individual differences in implicit attitudes, stereotypes, and identity are associated with variation in behavior toward individuals as a function of their social group membership” (p. 570). The second author coauthored an article with the claim that “Black participants’ implicit attitudes reflected no ingroup/outgroup preference . . . Black participants’ explicit attitudes reflected an ingroup preference” (Jiang et al., 2019). In 2007, Cunningham wrote that the “distinction between automatic and controlled processes now lies at the heart of several of the most influential models of evaluative processing” (Cunningham & Zelazo, 2007, p. 97). And Cunningham coauthored a review article with the claim that “a variety of tasks have been used to reflect implicit psychopathology associations, with the IAT (Greenwald et al., 1998) used most widely” (Teachman et al., 2019). Finally, many users of IATs assume that they are measuring implicit constructs that are distinct from constructs that are measured with self-ratings. It is therefore a problem for the construct validity of IATs if they lack discriminant validity. At the least, Kurdi et al. fail to explain why anybody should use IATs if they merely measure the same constructs that can be measured with cheaper self-ratings. In short, the question whether IATs and explicit measures reflect the same constructs or different constructs has theoretical and empirical relevance, and lack of discriminant validity is a problem for many theories of implicit cognitions (but see Cunningham & Zelazo, 2007).

### False fact 2

A more serious false claim is that I found “high correlations between relatively indirect (automatic) measures of mental content, as indexed by the IAT, and relatively direct (controlled) measures of mental content, as indexed by a variety of self-report scales” (p. 423). Table 2 shows some of the correlations among implicit and explicit measures in Vianello and Bar-Anan’s data. Only one of these correlations meets the standard criterion of a high correlation (i.e., *r* = .5; Cohen, 1988). The other correlations are small to moderate. These correlations show at best moderate convergent validity and no evidence of discriminant validity (i.e., higher implicit-implicit than implicit-explicit correlations). Similar results have been reported since the first IATs were created (Bosson et al., 2000). For 20 years, IAT researchers have ignored these low correlations and made grand claims about the validity of IATs. Kurdi et al. are doubling down on this misinformation by falsely describing these correlations as high.

**Table 2.** Correlations for Self-Ratings, IATs, and Evaluative Priming

Construct	Self-Rating–Implicit Association Test	Self-Rating–Evaluative Priming	Implicit Association Test–Evaluative Priming
Race	.31	.15	.24
Political orientation	.63	.39	.38
Self-esteem	.13	−.05	.02

Note: Data are from Vianello and Bar-Anan (2021).

### False fact 3

The third false claim is that “plenty of evidence in favor of dissociations between direct and indirect measures exists” (p. 428). To support this claim, Kurdi et al. cite a meta-analysis of incremental predictive validity (Kurdi et al., 2019). There are several problems with this claim.

First, the meta-analysis corrects only for random measurement error and not systematic measurement error. To the extent that systematic measurement error is present, incremental validity will shrink because explicit and implicit factors are very highly correlated when both sources of error are controlled (Schimmack, 2021). Second, Kurdi et al. fail to mention effect sizes. The meta-analysis suggests that a perfectly reliable IAT would explain about 2% unique variance. However, IATs have only modest reliability. Thus, manifest IAT scores would explain even less unique variance.

Finally, even this estimate has to be interpreted with caution because the meta-analysis did not correct for publication bias and included some questionable studies. For example, Phelps et al. (2003) report, among 12 participants, a correlation of .58 between scores on the race IAT and differences in amygdala activation in response to Black and White faces. Assuming 20% valid variance in the IAT scores (Schimmack, 2021), the validation-corrected correlation would be 1.30. In other words, a correlation of .58 is impossible given the low validity of race-IAT scores. It is well known that correlations in functional MRI studies with small samples are not credible (Vul et al., 2009). Moreover, brain activity is not a social behavior. It is therefore unclear why studies like this were included in Kurdi et al.’s (2019) meta-analysis.

Kurdi et al. also used suicides as an important outcome that can be predicted with suicide and death IATs. They cited two articles to support this claim. Fact checking shows that one article reported a statistically significant result (*p* = .013; Barnes et al., 2017), whereas the other one did not (*p* > .50; Glenn et al., 2019). I conducted a meta-analysis of all studies that reported incremental predictive validity of suicide or death IATs. The criterion was suicide attempts in the next 3 to 6 months (Table 3). I found eight studies, but six of them came from a single lab (Matthew K. Nock). Nock was also the first one to report a significant result in an extremely underpowered study that included only two suicide attempts (Nock & Banaji, 2007). Five of the eight studies showed a statistically significant result (63%), but the average observed power to achieve significance was only 42%. This discrepancy suggests the presence of publication bias (Schimmack, 2012). Moreover, significant results are all clustered around .05, and none of the *p* values meets the stricter criterion of .005 that has been suggested by Nosek and others to claim a discovery (Benjamin et al., 2018). Thus, there is no conclusive evidence to suggest that suicide IATs have incremental predictive validity in the prediction of suicides. This is not surprising because most of the studies were underpowered and unlikely to detect small effects. Moreover, effect sizes are bound to be small because the convergent validity between suicide and death IATs is low (*r* = .21; Chiurliza et al., 2018), suggesting that most of the variance in these IATs is measurement error.

**Table 3.** Meta-Analysis of Suicide/Death IATs Incremental Predictive Validity

Author	Year	*N*	Attempts	*p*	*z*	Sig.	Pow	Inflation	R index
Nock	2007	73	2	.03	2.14	1	0.57	0.43	0.14
Nock	2010	157	14	.04	2.03	1	0.53	0.47	0.06
Randall	2013	107	29	.02	2.32	1	0.64	0.36	0.28
Barnes	2017	163	27	.01	2.46	1	0.69	0.31	0.38
Harrison	2018	128	23	.41	0.82	0	0.13	−0.13	0.25
Glenn	2019	131	6	.53	0.62	0	0.09	−0.09	0.18
Millner	2019	71	5	1.00	0.00	0	0.02	−0.02	0.05
Tello	2019	164	16	.01	2.47	1	0.70	0.30	0.39
Average	—	124	15	.11^a^	1.61	.63	0.42	0.20	0.22

Note: Attempts = number of participants with suicide attempts, Sig. = significant (H0 was rejected), Pow = observed power, Inflation = sig – power, R-index = replicability index (power – inflation)

a This value is based on the mean *z* score.

In conclusion, 20 years of research with IATs has produced no credible and replicable evidence that IATs have incremental predictive validity over explicit measures. Even if there is some statistically significant incremental predictive validity, the amount of explained variance may lack practical significance (Kurdi et al., 2019).

### False fact 4

Kurdi et al. (2021) object (p. 424) to my claim that “most researchers regard the IAT as a valid measure of enduring attitudes that vary across individuals” (Schimmack, 2021, p. 397). They claim that “the overwhelming theoretical consensus in the community of attitude researchers. . . is that attitudes emerge from an interaction of persons and situations” (p. 425). It is instructive to compare this surprising claim with Cunningham and Zelazo’s (2007) definition of attitudes as “relatively stable ideas about whether something is good or bad” (p. 97). Kurdi and Banaji (2017) wrote that “differences in implicit attitudes . . . may arise because of multiple components, *including relatively stable components* [emphasis added]” (p. 286). Rae and Greenwald (2017) stated that it is a “widespread assumption . . . that implicit attitudes are characteristics of people, almost certainly more so than a property of situations” (p. 297). Greenwald and Lai (2020) stated that test–retest reliability “places an upper limit on correlational tests of construct validity” (p. 425). This statement makes sense only if we assume that the construct to be measured is stable over the retest interval.

It is also not clear how it would be ethical to provide individuals with feedback about their IAT scores on the Project Implicit website, if IAT scores were merely a product of the specific situation at the moment they are taking the test. Finally, how can the suicide IAT be a useful predictor of suicide if it cannot not measure some stable dispositions related to suicidal behaviors? In conclusion, Kurdi et al.’s definition of attitudes is inconsistent with the common definition of attitudes as relatively enduring evaluations.

That being said, the more important question is whether IATs measure stable attitudes or momentary situational effects. Ironically, some of the best evidence comes from Cunningham. Cunningham et al. (2001) repeatedly measured prejudice four times over a 3-month period with multiple measures, including the race IAT. Cunningham et al. (2001) modeled the data with a single trait factor that explained all of the covariation among different measures of racial attitudes. Thus, Cunningham et al. (2001) provided first evidence that most of the valid variance in race IAT scores is perfectly stable over a 3-month period and that person-by-situation interactions had no effect on racial attitudes.

There have been few longitudinal studies with IATs since Cunningham et al.’s (2001) seminal study. However, last year, an article examined stability over a 6-year interval (Onyeador et al., 2020). Racial attitudes of more than 3,000 medical students were measured in the first year of medical school, the fourth year of medical school, and the second year of medical residency. Table 4 shows the correlations for the explicit feeling thermometer and the IAT scores. The first observation is that the Time-1-to-Time-3 correlation for the IAT scores is not smaller than the Time-1-to-Time-2 or the Time-2-to-Time-3 correlations. This pattern shows that a single trait factor can capture the shared variance among the repeated IAT measures. The second observation is that the bold correlations between explicit ratings and IAT scores on the same occasion are only slightly higher than the correlations for different measurement occasions. This finding shows that there is very little occasion-specific variance in racial attitudes. The third observation is that IAT correlations over time are higher than the corresponding FT-IAT correlations over time. This finding points to IAT-specific method variance that is revealed in studies with multiple implicit measures (Cunningham et al., 2001; Schimmack, 2021). These findings extend Cunningham et al.’s (2001) findings to a 6-year period and show that most of the valid variance in race IAT scores is stable over long periods of time. In conclusion, Kurdi et al.’s claims about person-by-situation effects are not supported by evidence.

**Table 4.** Concurrent and Longitudinal correlations of implicit and explicit measures of prejudice

	FT1	FT2	FT3	IAT1	IAT2
FT1	—				
FT2	.52	—			
FT3	.43	.45	—		
IAT1	**.20**	.18	.18	—	
IAT2	.17	**.19**	.18	.37	—
IAT3	.18	.19	**.20**	.40	.40

Note: FT = feeling thermometer; IAT = implicit association test.

## Conclusion

Like presidential debates, the commentaries and my response present radically different views of reality. In one world, IATs are valid and useful tools that have led to countless new insights into human behavior. In the other world, IATs are noisy measures that add nothing to the information we already get from cheaper self-reports. Readers not well versed in the literature are likely to be confused rather than informed by these conflicting accounts. Although we may expect such vehement disagreement in politics, we should not expect it among scientists.

A common view of scientists is that they are able to resolve disagreement by carefully looking at data and drawing logical conclusions from empirical facts. However, this model of scientists is naive and wrong. A major source of disagreement among psychologists is that psychology lacks an overarching paradigm; that is, a set of fundamentally shared assumptions and facts. Psychology does not have one paradigm, but many paradigms. The IAT was developed within the implicit social-cognition paradigm that gained influence in the 1990s (Bargh et al., 1996; Greenwald & Banaji, 1995; Nosek et al., 2011). Over the past decade, it has become apparent that the empirical foundations of this paradigm are shaky (Doyen et al., 2012; D. Kahneman quoted in Yong, 2012, Supplemental Material; Schimmack, 2020). It took a long time to see the problems because paradigms are like prisons that make it impossible to see the world from the outside. A key force that prevents researchers within a paradigm from noticing problems is publication bias. Publication bias ensures that studies that are consistent with a paradigm are published, cited, and highlighted in review articles to provide false evidence in support for a paradigm (Greenwald & Lai, 2020; Kurdi et al., 2021).

Over the past decade, it has become apparent how pervasive these biases have been, especially in social psychology (Schimmack, 2020). The responses to my critique of IATs merely confirms how powerful paradigms and conflicts of interest can be. It is therefore necessary to allocate more resources to validation projects by independent researchers. In addition, validation studies should be preregistered and properly powered, and results need to be published whether they show validity or not. Conducting validation studies of widely used measures could be an important role for the emerging field of meta-psychology that is not focused on new discoveries, but rather on evaluating paradigmatic research from an outsider, meta-perspective (Carlsson et al., 2017). Viewed from this perspective, many IATs that are in use lack credible evidence of construct validity.

## Transparency

*Action Editor:* Laura A. King

*Editor:* Laura A. King

**ORCID iD:** Ulrich Schimmack  https://orcid.org/0000-0001-9456-5536
